# Stunting and growth velocity of adolescents with perinatally acquired HIV: differential evolution for males and females. A multiregional analysis from the IeDEA global paediatric collaboration

**DOI:** 10.1002/jia2.25412

**Published:** 2019-11-08

**Authors:** Julie Jesson, Michael Schomaker, Karen Malasteste, Dewi K Wati, Azar Kariminia, Mariam Sylla, Kouakou Kouadio, Shobna Sawry, Mwangelwa Mubiana‐Mbewe, Samuel Ayaya, Rachel Vreeman, Catherine C McGowan, Marcel Yotebieng, Valériane Leroy, Mary‐Ann Davies, the IeDEA global cohort consortium

**Affiliations:** ^1^ Inserm U1027 Université Paul Sabatier Toulouse 3 Toulouse France; ^2^ University of Cape Town Centre for Infectious Disease Epidemiology and Research Cape Town South Africa; ^3^ Medical Informatics and Technology Institute of Public Health UMIT ‐ University for Health Sciences Medical Decision Making and Health Technology Assessment Hall in Tirol Austria; ^4^ Inserm U1219 Bordeaux Population Health Center Université de Bordeaux Bordeaux France; ^5^ Sanglah Hospital Bali Indonesia; ^6^ The Kirby Institute UNSW Sydney Australia; ^7^ Hopital Gabriel Touré Bamako Mali; ^8^ CIRBA Abidjan Côte d’Ivoire; ^9^ Harriet Shezi Children’s Clinic Chris Hani Baragwanath Academic Hospital Soweto South Africa; ^10^ Faculty of Health Scences Wits Reproductive Health and HIV Institute University of the Witwatersrand Johannesburg South Africa; ^11^ Centre for Infectious Disease Research in Zambia Lusaka Zambia; ^12^ Department of Child Health and Paediatrics School of Medicine College of Health Sciences Moi University Eldoret Kenya; ^13^ Ryan White Center for Pediatric Infectious Disease and Global Health Department of Pediatrics Indiana University School of Medicine Indianapolis IN USA; ^14^ Vanderbilt University School of Medicine Nashville TN USA; ^15^ Division of Epidemiology College of Public Health The Ohio State University Columbus OH USA

**Keywords:** HIV, adolescent, growth, stunting, cohort studies, developing countries

## Abstract

**Introduction:**

Stunting is a key issue for adolescents with perinatally acquired HIV (APH) that needs to be better understood. As part of the IeDEA multiregional consortium, we described growth evolution during adolescence for APH on antiretroviral therapy (ART).

**Methods:**

We included data from sub‐Saharan Africa, the Asia‐Pacific, and the Caribbean, Central and South America regions collected between 2003 and 2016. Adolescents on ART, reporting perinatally acquired infection or entering HIV care before 10 years of age, with at least one height measurement between 10 and 16 years of age, and followed in care until at least 14 years of age were included. Characteristics at ART initiation and at 10 years of age were compared by sex. Correlates of growth defined by height‐for‐age z‐scores (HAZ) between ages 10 and 19 years were studied separately for males and females, using linear mixed models.

**Results:**

Overall, 8737 APH were included, with 46% from Southern Africa. Median age at ART initiation was 8.1 years (interquartile range (IQR) 6.1 to 9.6), 50% were females, and 41% were stunted (HAZ<−2 SD) at ART initiation. Males and females did not differ by age and stunting at ART initiation, CD4 count over time or retention in care. At 10 years of age, 34% of males were stunted versus 39% of females (*p* < 0.001). Females had better subsequent growth, resulting in a higher prevalence of stunting for males compared to females by age 15 (48% vs. 25%) and 18 years (31% vs. 15%). In linear mixed models, older age at ART initiation and low CD4 count were associated with poor growth over time (*p* < 0.001). Those stunted at 10 years of age or at ART initiation had the greatest growth improvement during adolescence.

**Conclusions:**

Prevalence of stunting is high among APH worldwide. Substantial sex‐based differences in growth evolution during adolescence were observed in this global cohort, which were not explained by differences in age of access to HIV care, degree of immunosuppression or region. Other factors influencing growth differences in APH, such as differences in pubertal development, should be better documented, to guide further research and inform interventions to optimize growth and health outcomes among APH.

## 1 Introduction

Adolescence, defined by the World Health Organization (WHO) as between 10 and 19 years of age 1, is a critical transition period in life, accompanied by significant biological and psychosocial changes 2. In the context of HIV, major improvements in access to antiretroviral therapy (ART) for children with perinatally acquired HIV infection have led to reductions in HIV‐related mortality, resulting in a growing population of adolescents living with perinatally acquired HIV (APH). In 2016, it was estimated that 2.1 million adolescents were living with HIV worldwide, with 80% in sub‐Saharan Africa 3. AIDS‐related deaths are one of the leading causes of mortality among adolescents in this region 4. Adolescents living with HIV face specific challenges in terms of HIV care and outcomes 5, including HIV disclosure issues 6, access to sexual and reproductive health services 7 and transition from paediatric to adult care 8. Adolescents are reported to have the lowest adherence to ART 9 and high rates of lost‐to‐follow‐up (LTFU) 10.

Growth deficiency is a key concern for children and adolescents living with HIV 11, 12. There are overlapping effects on growth resulting from a vicious cycle between malnutrition and HIV infection. Malnutrition impairs the immune system and weakens the body’s defences, leading to HIV disease progression 13; while HIV infection, related opportunistic infections and chronic inflammation may weaken nutritional status 14, 15. Repeated episodes of malnutrition during infancy and childhood may have occurred frequently among APH, and lifelong HIV infection can lead to chronic malnutrition and growth retardation as APH age into adulthood. Adolescence is the second most important period of growth after the first year of life 16, hence these factors may hinder pubertal growth spurt for APH. Chronic diseases, malnutrition and HIV infection during childhood could also delayed puberty, worsening growth retardation during adolescence for APH 17, 18, 19, 20.

While data show that nearly half of adolescents suffer from stunting in some developing countries 21, data on growth and stunting are limited among APH. Several studies have shown that growth improves after ART initiation, with larger increases for children initiated earlier on ART 22, 23, but most of these studies were conducted among children younger than 10 years of age or during the first two years on ART. Little is known about growth during adolescence among APH and those on long‐term ART. As part of the IeDEA (International epidemiology Databases to Evaluate AIDS) multiregional consortium, we described growth evolution and its associated factors during adolescence for males and females with perinatally acquired HIV.

## 2 Methods

### 2.1 Study population

The IeDEA global paediatric collaboration is part of the global IeDEA research consortium (https://www.iedea.org/), supported by the US National Institutes of Health since 2006, to describe HIV epidemiology and evaluate HIV outcomes using large patient‐level observational databases. HIV care cohorts from sub‐Saharan Africa (West, Central, Eastern and Southern Africa regions), the Asia‐Pacific, and the Caribbean, Central and South America (CCASAnet) regions contributed data for this analysis. We merged data collected from HIV clinics in partnership with IeDEA from these six sub‐regions between 2003 and 2016. Among patients who had at least one visit available during adolescence, with at least one height measurement during this period (i.e. age 10 to 19 years), inclusion criteria to assess growth throughout adolescence were as follows: reported perinatally acquired infection or entering HIV care before 10 years of age (proxy for perinatally acquired HIV when not documented); having a documented date of ART initiation, at least one height measurement between 10 and 16 years of age; and followed in care until at least 14 years of age. APH were thus excluded if known to have acquired HIV non‐perinatally or if not known, be enrolled in care after 10 years of age. They were then excluded if no ART initiation (excluded group A) and no HAZ measurements between 10 and 16 years of age (excluded group B) were documented, and if they were not followed after 14 years of age (excluded group C).

### 2.2 Variables and data management

We studied growth using height‐for‐age, defined by the WHO child growth standards 24, 25, expressed in z‐scores (HAZ). Stunting was defined as HAZ lower than − 2 standard deviations (SD), with moderate stunting between − 3 and − 2 SD and severe stunting lower than − 3 SD. Z‐scores lower than − 10 or greater than + 10 were viewed as outliers and removed. Growth velocity defined as height gain in centimetre (cm) per year was compared to the reference population from the WHO child growth standards for a subset of adolescents having at least two height measurements. We calculated growth velocity per year by dividing the height gain in cm between two clinical visits by the time in years between these two visits. Velocities greater than 20 cm/year and below − 1 cm/year were excluded as outliers. Wasting was defined using weight‐for‐height z‐score (WHZ) for children under five years of age, and body‐mass‐index‐for‐age z‐score (BAZ) for children aged more than five years. Immunodeficiency for age at ART initiation was defined following the 2006 WHO guidelines 26. The location of the clinic site (urban or mostly urban, rural or mostly rural) was also recorded. Follow‐up for patients was analysed on a six‐monthly basis, with data on immunologic, clinical, anthropometric status and ART regimen collected. Loss to follow‐up was defined as having a last contact longer than six months before database closure in children not known to have died or transferred out, and transfer was documented when adolescents were transferred to another paediatric clinic or to adult care.

### 2.3 Ethics approval

Each participating IeDEA region obtained local institutional review boards’ approvals to participate. Consent requirements were deferred to the local institutional review boards. The analysis only used anonymized data that had been collected as part of routine clinical care.

### 2.4 Statistical analysis

Patient characteristics were described at ART initiation and at age 10 years, and compared by sex using Chi‐square tests for categorical variables and Kruskal‐Wallis tests for continuous variables. Characteristics of the study population were also compared to those of excluded adolescents. Prevalence of stunting was measured at ART initiation and each year between 10 and 19 years of age, stratified by sex. Mean HAZ, together with 95% confidence intervals, was plotted stratified by age and sex.

The analysis and inclusion to the study started at age 10 years (baseline). Growth, modelled as mean HAZ between ages 10 and 19 years, was studied using linear mixed models, with an unstructured variance‐covariance matrix and random intercept. All participants with at least one HAZ were able to contribute to the model 27. The model contained time‐varying age (=10 years plus follow‐up time) as a covariate which was included non‐linearly using (penalized) splines 28. Age at ART initiation, stunting and wasting at ART initiation and at age 10 years, current CD4 count, CD4 count at age 10 years, CD4 count at ART initiation and at first visit as well as region and location of clinic site were considered as potential adjustment variables. To take into account missing data, especially at baseline, we conducted multiple imputation for stunting and wasting at age 10 years and ART initiation as well as CD4 count and location, using the Amelia II package in R 29. The imputation model contained all measured variables, as well as splines of time and lagged variables of CD4 count. Variable selection using the AIC criteria was applied to the imputed data sets 30, 31. The final mixed model containing the selected variables was fitted in all five imputed data sets and results were combined according to Rubin’s rules 32. The penalized spline for the association of age with HAZ is displayed for the first imputed data set (see Figure S1 for the other imputed data sets).

## 3 Results

### 3.1 Selection and characteristics of the study population

**Figure 1:**
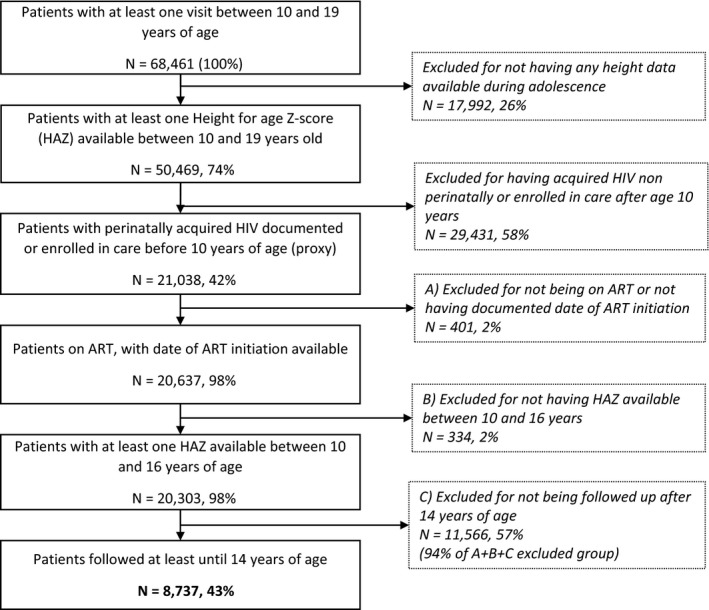
Flowchart of the study population, IeDEA global paediatric collaboration, 2003 to 2016.

Between 2003 and 2016, 68,461 patients receiving care in the six IeDEA regional cohorts had at least one visit during adolescence. Of these, 50,469 (74%) had one available HAZ measurement during the same period, and 21,038 (42%) of them met criteria for perinatally acquired infection. Among APH on ART, the majority had at least one available HAZ measurement between 10 and 16 years of age. The median age at last follow‐up was 14.2 (interquartile range (IQR) 12.1 to 16.5 years), with 43% followed up until at least 14 years of age. Among the APH excluded for not being followed after 14 years of age, 73% had not yet reached age 14 years by the time the database was closed. The final study population included 8737 APH (Figure 1).

APH excluded for three criteria (no information on ART initiation (A), no HAZ measurements between 10 and 16 years of age (B), no follow‐up after 14 years of age (C), Figure 1) were compared to the final study population. Those from the exclusion group A (n = 401) were more frequently LTFU (22% vs. 8%) and less stunted (18% vs. 29%, *p* < 0.001). The exclusion group B (n = 334) initiated ART mostly after age 10 years (63% vs. 17%, *p* < 0.001), with a higher rate of death (4% vs. 2%, *p* = 0.003) and LTFU (39% vs. 8%, *p* < 0.001). Finally, the exclusion group C (n = 11,566) was more likely to have started ART below five years of age compared to the study population (34% vs. 15%, *p* < 0.001), they more frequently had missing data for assessing stunting, wasting and CD4 count at 10 years (*p* < 0.001) and had slightly higher rates of LTFU (10% vs. 8%, *p* < 0.001) but mortality was similar. The latter group comprised 94% of those excluded for these three reasons and are mostly children who had not yet reached the age of 14 years as they had started ART more recently and at younger ages and could not contribute to examining growth throughout adolescence (Table S1).

Overall, 50.5% were females, 45.6% were living in Southern Africa, and 76.7% were followed in an urban or mostly urban clinical care centre. Median age at ART initiation was 8.1 years (IQR 6.1 to 9.6), with 84.8% initiating ART after age five years. The prevalence of stunting at ART initiation was 49.6% among those with available HAZ measurement (n = 6560); of these, 45.9% were severely stunted. Among those with available WHZ/BAZ data (n = 7103), 18% were wasted at ART initiation and 10% at age 10 years. Overall, characteristics at ART initiation did not differ by sex, with similar age groups (*p* = 0.186), CD4 count (*p* = 0.063) and stunting (*p* = 0.101). Males were slightly more wasted than females at ART initiation (*p* = 0.010). The absolute differences in median age at ART initiation (males vs. females: 8.02 vs. 8.19, *p* = 0.007) and median HAZ (males vs. females: −2.05 vs. −1.96, *p* = 0.020) were minimal (Table 1). At age 10 years, the prevalence of stunting was 34.4% among males and 39.5% among females (*p* < 0.001), with about one‐third severely stunted in each group. There were no observed differences by sex in CD4 count groups or the severity of wasting (Table 1).

**Table 1:** Characteristics at ART initiation and at 10 years of age for males and females, N = 8737, IeDEA global paediatric collaboration, 2003 to 2016

Variables	Males (N = 4329)		Females (N = 4408)		*p*‐value^a^	Total	
*Characteristics at ART initiation*							
Age							
Median, IQR (years)	8.0 [6.1 to 9.5]		8.2 [6.2 to 9.6]		0.007	8.1 [6.1 to 9.6]	
Age groups					0.186		
0 to 2 years	146	3.4	166	3.8		312	3.6
2 to 5 years	527	12.2	488	11.1		1015	11.6
5 to 10 years	2950	68.1	2959	67.1		5909	67.6
>10 years	706	16.3	795	18.0		1501	17.2
CD4 count							
Median, IQR (cell/mm^3^)	294 [130 to 517]		307 [148 to 516]			301 [139 to 516]	
Immunodeficiency for age^b^					0.148		
No	604	13.9	643	14.6		1247	14.3
Moderate	1008	23.3	1087	24.7		2095	24.0
Severe	1598	36.9	1620	36.7		3218	36.8
Missing	1119	25.9	1058	24.0		2177	24.9
HAZ							
Median, IQR (z‐score)	−2.05 [−2.95 to −1.16]		−1.96 [−2.85 to −1.05]		0.020	−1.99 [−2.90 to −1.11]	
Stunting groups					0.101		
No stunting	1742	40.2	1873	42.5		3615	41.4
Moderately stunted	949	21.9	974	22.1		1923	22.0
Severely stunted	837	19.3	795	18.0		1632	18.7
Missing	801	18.5	766	17.4		1567	17.9
WHZ/BAZ							
Wasting groups					0.010		
No wasting	2828	65.3	3010	68.3		5838	66.8
Moderately wasted	362	8.4	346	7.8		708	8.1
Severely wasted	306	7.1	251	5.7		557	6.4
Missing	833	19.2	801	18.2		1634	18.7
*Characteristics at 10 years*							
CD4 count							
Median, IQR (cell/mm^3^)	652 [410 to 920]		674 [434 to 952]		0.008	664 [423 to 938]	
CD4 count groups					0.133		
<250	297	6.9	272	6.2		569	6.5
>250	2292	52.9	2420	54.9		4712	53.9
Missing	1740	40.2	1716	38.9		3456	39.6
HAZ							
Median, IQR (Z‐score)	−1.59 [−2.32 to −0.87]		−1.70 [−2.48 to −0.90]		<0.001	−1.64 [−2.41 to −0.89]	
Stunting groups					<0.001		
No stunting	2221	51.3	2079	47.2		4300	49.2
Moderately stunted	854	19.7	900	20.4		1754	20.1
Severely stunted	310	7.2	460	10.4		770	8.8
Missing	944	21.8	969	22.0		1913	21.9
WHZ/BAZ							
Wasting groups					0.233		
No wasting	3109	71.8	3179	72.1		6288	72.0
Moderately wasted	259	6.0	257	5.8		516	5.9
Severely wasted	122	2.8	95	2.2		217	2.5
Missing	839	19.4	877	19.9		1716	19.6
*Context*							
Location of clinical centres							
Urban or mostly urban	3321	76.7	3438	78.0	0.121	6759	77.4
Rural or mostly rural	947	21.9	895	20.3		1842	21.1
Missing	61	1.4	75	1.7		136	1.6
Regions					0.022		
West Africa	361	8.3	345	7.8		706	8.1
Central Africa	200	4.6	200	4.5		400	4.6
East Africa	741	17.1	752	17.1		1493	17.1
Southern Africa	2028	46.9	1956	44.4		3984	45.6
Asia‐Pacific	724	16.7	817	18.5		1541	17.6
CCASAnet	275	6.4	338	7.7		613	7.0

BAZ, BMI‐for‐Age Z‐score, WHO Child Growth Standards; HAZ, Height‐for‐Age Z‐score; IQR, interquartile range; WHZ, Weight‐for‐Height Z‐score.

a Comparison tests between males and females using chi square and Kruskal‐Wallis tests.

b WHO 2006 guidelines.

Median number of height measurements was 21 (IQR 11 to 33). Males and females had similar retention, with a median age at enrolment (clinic entry) of 7.1 years (IQR 5.1 to 8.6, *p* = 0.425) and median age at last visit of 16.1 years (IQR 14.9 to 17.7, *p* = 0.160); 16.7% were transferred (*p* = 0.435), 7.6% were LTFU (*p* = 0.966) and 1.9% died during adolescence (*p* = 0.283) (data not shown).

### 3.2 Growth development during adolescence

#### 3.2.1 Growth velocity curves

**Figure 2:**
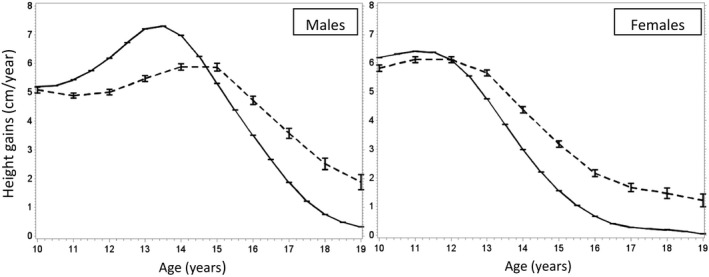
Mean height gain in cm per year for males (left) and females (right) during adolescence (dashed line), compared to the reference population of the WHO child growth standards (solid line). IeDEA global paediatric collaboration, 2003 to 2016.

In patients with at least two height measurements (n = 6397), growth velocity during adolescence was highest at age 13 years for APH males (mean value: 7.2 cm per year) while the peak growth velocity was reached at age 11 for APH females (mean value: 6.4 cm per year). Overall, growth velocity among males was similar to the reference population at age 10 but then diverged with relative reductions among APH until age 15 (mean value at age 13 years: 5.5 cm per year). After this period, while growth velocity for the reference population decreased, it stayed at a higher level for APH throughout late adolescence (mean values: 4.7 vs. 3.5 cm per year at 16 years, 1.9 vs. 0.3 cm per years at 19 years). For females, growth velocity was slightly lower than reference values for APH until age 12 years (mean value at 11 years: 6.1 cm per year) and then stayed consistently higher (means values: 3.2 vs. 1.6 cm per year at 15 years, 1.7 vs. 0.3 cm per year at 17 years, 1.2 vs. 0.1 cm per year at 19 years) (Figure 2).

#### 3.2.2 Prevalence of stunting and HAZ curves

**Figure 3:**
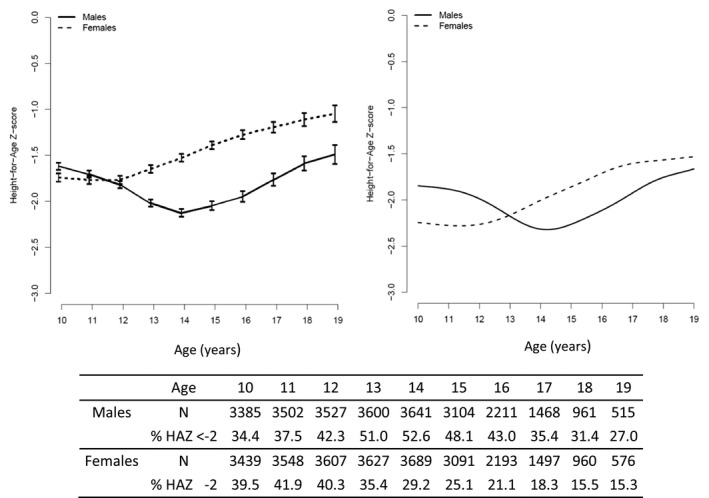
Mean Height‐for‐Age Z‐score (HAZ) evolution during adolescence with sample size and prevalence of stunting at each age (N = 8737). Crude results (left) and adjusted estimates of the first imputed mixed model (right) using the following reference population: ART start at age 5, current CD4 = 100, CD4 at age 10 = 100, not rural, moderate stunting at age 10 and ART start, region Asia‐Pacific. IeDEA global paediatric collaboration, 2003 to 2016.

HAZ evolution during adolescence differed by sex and was in line with the deviations observed in growth velocity. For males, the growth deficit observed in early adolescence resulted in a decrease of mean HAZ between 10 and 14 years of age (mean values: −1.6 SD at 10 years, −2.1 SD at 14 years), followed by a constant increase until age 19 years, reaching similar values to those at age 10 (mean value: −1.43 SD at 19 years). For females, mean HAZ increased continuously during adolescence, especially after age 12 years (mean values: −1.7 SD at 12 years, −1.3 SD at 15 years, −1.0 SD at 19 years). HAZ was higher for females than males after age 12 years, leading to a higher prevalence of stunting for males (51% vs. 35% at 13 years, *p* < 0.001; 31% vs. 15% at 18 years, *p* < 0.001) (Figure 3, left‐hand side). The imputed mixed model resulted in adjusted HAZ estimates close to the crude values (Figure 3, right‐hand side). Trends in growth evolution by sex were similar by region (Figure S2).

#### 3.2.3 Factors associated with HAZ evolution

Multivariable imputed linear mixed models are shown in Table 2. Most of the factors associated with HAZ evolution were common for both males and females. Older age at ART initiation was associated with a lower mean HAZ over time (example for males: mean difference of −0.149 SD for each year over time, *p* < 0.001). Higher CD4 count over time was associated with higher HAZ (example for females: +0.019 SD per 100 cells/mm^3^). Those with moderate or severe stunting at ART initiation had lower mean HAZ at baseline (*p* < 0.001) but also higher HAZ increase between 10 and 19 years of age compared to those not stunted (*p* < 0.001). For males, growth evolution also depended on CD4 count at baseline (+0.003 SD per 100 cells/mm^3^ over time). Males who were followed in rural HIV clinic centres had lower HAZ increase compared to those followed in urban centers (−0.047 SD per year, *p* < 0.001), while mean HAZ was similar at 10 years of age (*p* = 0.289).

**Table 2:** Factors associated with HAZ evolution between 10 and 19 years of age for males and females

Variables	Males	*p*‐value	Differences in yearly mean (SD) HAZ increase	*p*‐value	Females	*p*‐value	Differences in yearly mean (SD) HAZ increase	*p*‐value
	Differences in mean (SD) HAZ at age 10 or over time				Differences in mean (SD) HAZ at age 10 or over time			
Age at ART initiation (years)	−0.149 (−0.019)	<0.001			−0.113 (0.017)	<0.001		
Current CD4 count (per 100 cells/mm^3^)	0.014 (0.001)	<0.001			0.019 (0.004)	0.004		
CD4 count at 10 years (per 100 cells/mm^3^)	0.003 (0.001)	0.005			–	–		
Stunting at ART initiation								
Moderate versus no	−0.322 (0.022)	<0.001	0.018 (0.038)	<0.001	−0.307 (0.024)	<0.001	0.023 (0.003)	<0.001
Severe versus no	−0.229 (0.018)	<0.001	0.013 (0.002)	<0.001	−0.255 (0.018)	<0.001	0.013 (0.002)	<0.001
Stunting at 10 years of age								
Moderate versus no	−0.543 (0.028)	<0.001	0.007 (0.009)	<0.001	−0.909 (0.023)	<0.001	0.127 (0.003)	<0.001
Severe versus no	−0.306 (0.018)	<0.001	−0.007 (0.006)	0.234	−0.639 (0.021)	<0.001	0.088 (0.002)	<0.001
Located in rural versus urban area	0.059 (0.045)	0.289	−0.047 (0.003)	<0.001	−0.010 (3.831)	0.795	–	–

Moderate stunting = (−3; −2 (SD, Severe stunting <−3 SD)). Multivariable linear mixed models. N = 8737, IeDEA global paediatric collaboration, 2003 to 2016. Adjusted on IeDEA regions. Interaction with time was added for the variables “stunting” for both males and females and location for males. Difference in estimates were thus expressed in mean HAZ at baseline (10 years of age) and in yearly mean HAZ increase (second column) for these variables. The other variables (age at ART initiation, current CD4 count and CD4 count at 10 years for both males and females and location for females) were expressed in difference in estimates over time (first column). Estimates for time variables: Males: t = −0.203, t^2^ = −0.014, t^3^ = −0.001; females: t = −0.149, t^2^ = 0.053, t^3^ = −0.004.

## 4 Discussion

Growth retardation represents a major concern for APH in IeDEA cohorts; half of APH were stunted at ART initiation, and the prevalence of stunting remained high during adolescence. While males and females had similar baseline and follow‐up characteristics, their growth patterns during adolescence diverged considerably. Despite males experiencing greater height gains later in adolescence, these were not sufficient to compensate for the smaller than expected height gains in early adolescence, resulting in persistently high rates of stunting until 19 years of age. Females had substantial height gains in mid‐ to late adolescence, above the population reference, allowing them to have a slow but constant HAZ increase throughout adolescence; resulting in a much lower prevalence of stunting by 19 years of age.

Few studies on growth have been conducted among APH, most of them focusing on the first years of ART 17. Some cross‐sectional studies have described stunting among adolescents with HIV. In West and Central Africa, among an ART‐treated population, the prevalence of stunting was 34% among patients aged 10 to 19 years 33 and 42% among patients aged 10 to 16 years in Senegal 34. In El Salvador, 48% of children with HIV over 12 years of age were stunted 35. In the Collaborative Initiative for Paediatric HIV Education and Research (CIPHER) including cohorts of APH from IeDEA and other networks in low‐, middle‐ and high‐income countries, median HAZ at ART initiation and at 10 years of age were similar to our study; however, APH living in high‐income countries had higher HAZ than in low‐and‐middle‐income countries (LMICs) 36.

In our cohort, late age at ART initiation was strongly associated with stunting and poor HAZ evolution. This finding has been observed in many other studies, but mostly among children younger than age 10 years 22, 23. Children who spend their first years of life with HIV without ART may have chronic inflammation due to uncontrolled HIV, which can affect growth. The longer this exposure to uncontrolled HIV, the more adversely growth might be affected. Repeated opportunistic infections like pneumonia, chronic diarrhoea and tuberculosis during this period can also result in stunting. It is concerning that despite the WHO recommendations for immediate ART initiation, age at ART initiation was high, approximately a year later than age at enrolment. This population likely had poor access to ART, as already highlighted by the IeDEA collaboration 37. While reasons for delayed ART initiation should be examined, most of these children would have first enrolled before the WHO recommendation for immediate ART in all children < 15 years of age, and hence would have had to meet disease severity or CD4 criteria before initiating ART. With better access to paediatric care and earlier ART initiation than during the study period for children living with HIV, we could expect to observe lower rates of stunting at ART initiation and better catch‐up growth during adolescence in the future, decreasing the burden of stunting for APH. However, stunting is still highly prevalent in most of the study settings in the general population and access to early ART initiation is not yet a reality in some sub‐regions such as in West and Central Africa.

Similarly, adolescents with low CD4 count over time had poorer growth in our cohort. Even on ART, chronic immunodeficiency could alter growth 13. We observed a trend of poorer growth evolution among males attending rural clinics, which suggests higher vulnerability of APH living in rural settings that could be related to poverty and food access as well as the general challenges of ART adherence and retention in HIV care observed during adolescence 37.

The growth differences observed between males and females in this study were not fully explained by the available data. The pubertal development of APH could be different for males and females and have varying effects on growth, but the literature on this topic is scarce 17, 19, 38, especially in LMICs 18. In the US, compared to HIV‐exposed but uninfected adolescents, APH had delayed pubertal onset, from six to eight months later in females and 10 to 11 months later in males. In that study, however, the mean HAZ value was normal and there were no apparent links between pubertal development and low HAZ 17. Ugandan and Zimbabwean APH have been reported to have substantial pubertal delays, more pronounced for those who were stunted and initiated ART late, but similar between males and females, although males faced more height disadvantage throughout adolescence, as in our study 18.

Another possible explanation for the growth differences by sex was found in a US study where APH males had lower bone mineral density (BMD) compared to males without HIV in late puberty, while there were no differences for females. However, there were no height differences between those with and without HIV in this study 39. To the best of our knowledge, the effect of BMD on growth among adolescents living in LMICs has not been explored 40.

Delayed pubertal onset is also observed in the general population in LMICs 41, and may reflect poor nutrition during the first 1000 days of life and in later childhood 42, which is often the case for children with HIV 22. In rural villages of The Gambia, HAZ evolution was described from birth to 30 years and showed similar patterns to our study, with an apparent decline at the start of adolescence, especially for males, which could be explained by high rates of stunting during infancy and a later entry into puberty compared to the UK reference population 43. Without further data on the timing of puberty onset and hormonal pathways in children with HIV, it is difficult to determine the links and the causal relationships between delayed pubertal onset and growth retardation during adolescence, for males and females.

The trajectory of growth evolution was similar across the IeDEA regions, with differences between males and females. The hypothesis that in some socio‐economic and cultural contexts males could be disadvantaged compared to females regarding nutrition and access to food is therefore unlikely to explain our results. Nutritional habits among adolescents are however insufficiently documented in the general population in our contexts, and even less well documented for adolescents with HIV 43.

Our study has some limitations. Besides lacking puberty data like Tanner staging or age at first menstruation, we also did not have detailed anthropometric data around birth and the first 1000 days of life to explain some aspects of future growth 21. There were insufficient data on HIV viral load to assess the potential association with HAZ. A study in Botswana showed that severe short stature among children and adolescents with HIV was associated with virologic failure 44. In a review on growth after ART initiation among children, the ART regimen used was not associated with growth evolution 22. Also, pregnancy data were not recorded in all regions and then not included. Metabolic data were also not available, but would have been valuable to better assess the effect of long‐term ART on growth 45, 46

Our results could have been affected by selection bias, as they were driven by the large proportion of children from Southern Africa, which may not be representative of growth among APH worldwide. Excluding children who were not followed‐up until at least 14 years of age substantially restricted our sample size and adolescents who were LTFU or died before age 14 were not included, leading to survivor bias, with the risk of underestimating the prevalence of stunting in this population. Indeed, some studies tracing children after being LTFU have shown that this population was at higher risk of being severely immunodeficient and malnourished 47, 48. Furthermore, perinatal infection was defined using a threshold of enrolment in HIV care before age 10 years as a proxy, which excludes the subset of APH who may enter care after age 10 years and would be at even higher risk of stunting due to the delay in diagnosis, HIV care and ART initiation. Thus, the growth pattern described here may underestimate the full extent of stunting among APH.

We did not analyse outcomes beyond 19 years of age in order to focus on the period of adolescence. Additional height gains after age 19 might be expected, and thus this analysis might have concluded before we knew the final height attainments of all patients included. In rural non‐HIV populations in the Gambia, similar to our results, growth continued in late adolescence, and extended to the age of 22 to 24 years in males and 18 to 19 years in girls 43. This could be due to population‐level maturational delays, caused by delayed puberty onset, allowing stunted children and adolescents to catch‐up on their growth by prolonging pubertal growth 43, 49, with different timing in males and females 50. This mechanism highlights adolescence as a second critical window for growth after the first year of life, and the opportunity for catch‐up growth in stunted children living in LMICs, regardless of HIV status 42.

## 5 Conclusions

This study is, to our knowledge, the first multiregional study assessing growth among a large sample size of APH in LMICs. The high burden of stunting we observed could have serious consequences for social and cognitive development 51. Overall, APH are a vulnerable group, with multiple and interrelated HIV‐specific and general health needs 52. While males seem even more affected by stunting, it is important to ensure that all APH receive comprehensive support that addresses growth beyond childhood and into adolescence. Improved documentation of growth, nutritional status and pubertal phase among APH would provide key data to guide further research and inform interventions to optimize growth and health outcomes.

## Competing interest

Authors have no conflict of interest to declare.

## Funding

Research reported in this publication was supported by the US National Institutes of Health. Asia‐Pacific: The TREAT Asia Pediatric HIV Observational Database is an initiative of TREAT Asia, a program of amfAR, The Foundation for AIDS Research, with support from the US National Institutes of Health’s National Institute of Allergy and Infectious Diseases, the Eunice Kennedy Shriver National Institute of Child Health and Human Development, National Cancer Institute, National Institute of Mental Health, and National Institute on Drug Abuse as part of the International Epidemiology Databases to Evaluate AIDS (IeDEA; U01AI069907). Caribbean, Central and South America network for HIV epidemiology (CCASAnet) is a member cohort of the International Epidemiology Databases to Evaluate AIDS (leDEA) (U01AI069923; CMG is a PI). Central Africa research reported in this publication was supported by the National Institute of Allergy and Infectious Diseases of the National Institutes of Health under Award Number U01AI096299. East African Research reported in this publication was supported by the National Institute Of Allergy And Infectious Diseases (NIAID), Eunice Kennedy Shriver National Institute Of Child Health & Human Development (NICHD), National Institute On Drug Abuse (NIDA), National Cancer Institute (NCI), and the National Institute of Mental Health (NIMH), in accordance with the regulatory requirements of the National Institutes of Health under Award Number U01AI069911 (KWK is a PI), East Africa IeDEA Consortium. Southern African research reported in this publication was supported by the National Institute Of Allergy And Infectious Diseases of the National Institutes of Health under Award Number U01AI069924 (MAD is a PI). West African Research reported in this publication was supported by the US National Institutes of Health (NIAID, NICHD, NCI and NIMH) under Award Number U01AI069919. The funders had no role in study design, data collection and analysis, decision to publish, or preparation of the manuscript. Julie Jesson was funded by Sidaction.

## Authorship

JJ and M Schomaker conducted the analysis. JJ wrote the paper. MD, VL and M Schomaker supervised the analysis and the writing of the paper. KM contributed to the data management. DW and AK contributed and represent the cohort data from the Asia‐Pacific region, SS and MM for the Southern Africa region, M Sylla and KK for the Western Africa region, SA and RV from the Eastern Africa region, CM for CCASAnet and MY for Central Africa region. All authors have read and approved the final manuscript.

## Supporting information


**Table S1.** Comparison of characteristics between the study population and the excluded population according to three criteria.
**Figure S1.** Penalized splines for the association of age with HAZ for the 2 to 5 imputed data sets.
**Figure S2.** Mean Height‐for‐Age Z‐score (HAZ) evolution for males (blue) and females (red) during adolescence, trends by regions using raw data. IeDEA global pediatric collaboration, 2003 to 2016
