# Supplementary material for: Stunting and growth velocity of adolescents with perinatally acquired HIV: differential evolution for males and females. A multiregional analysis from the IeDEA global paediatric collaboration
Source: J Int AIDS Soc. 2019 Nov 8;22(11):e25412. doi: 10.1002/jia2.25412 (PMC6839428; doi:10.1002/jia2.25412)
Supplement: Supplementary file 1 — Table S1. Comparison of characteristics between the study population and the excluded population according to three criteria. Figure S1. Penalized splines for the association of age with HAZ for the 2 to 5 imputed data sets. Figure S2. Mean Height‐for‐Age Z‐score (HAZ) evolution for males (blue) and females (red) during adolescence, trends by regions using raw data. IeDEA global pediatric collaboration, 2003 to 2016 [file JIA2-22-e25412-s001.docx]

Table S1: Comparison of characteristics between the study population and the excluded population according to three criteria.

| **Characteristics** | **Included**  **N=8737** | |  | **Excluded A:**  **not on ART**  **N=401** | | **p-value** |  | **Excluded B:**  **no HAZ between**  **age 10-16y, N=334** | | **p-value** |  | **Excluded C:**  **no follow-up after**  **age 14y, N=11 566** | | **p-value** |
| --- | --- | --- | --- | --- | --- | --- | --- | --- | --- | --- | --- | --- | --- | --- |
| **Sex** |  |  |  |  |  | 0.012 |  |  |  | <0.001 |  |  |  | 0.330 |
| Females | 4408 | 50.5 |  | 228 | 56.9 |  |  | 206 | 61.7 |  |  | 5756 | 49.8 |  |
| Males | 4329 | 49.5 |  | 173 | 43.1 |  |  | 128 | 38.3 |  |  | 5811 | 50.2 |  |
| **Age at ART initiation** |  |  |  |  |  |  |  |  |  | <0.001 |  |  |  | <0.001 |
| 0-5y | 1327 | 15.2 |  | / | / |  |  | 8 | 2.4 |  |  | 3897 | 33.7 |  |
| 5-10y | 5909 | 67.6 |  | / | / |  |  | 117 | 35.0 |  |  | 6721 | 58.1 |  |
| >10y | 1501 | 17.2 |  | / | / |  |  | 209 | 62.6 |  |  | 948 | 8.2 |  |
| **Region** |  |  |  |  |  | <0.001 |  |  |  | <0.001 |  |  |  | <0.001 |
| West Africa | 706 | 8.1 |  | 56 | 14.0 |  |  | 147 | 44.0 |  |  | 1090 | 9.4 |  |
| Central Africa | 400 | 4.6 |  | 104 | 25.9 |  |  | 49 | 14.7 |  |  | 452 | 3.9 |  |
| East Africa | 1493 | 17.1 |  | 127 | 31.7 |  |  | 19 | 5.7 |  |  | 2313 | 20.0 |  |
| Southern Africa | 3984 | 45.6 |  | 0 | 0.0 |  |  | 71 | 21.3 |  |  | 6284 | 54.3 |  |
| Asia-Pacific | 1541 | 17.6 |  | 93 | 23.2 |  |  | 6 | 1.8 |  |  | 1152 | 10.0 |  |
| CCASAnet | 613 | 7.0 |  | 21 | 5.2 |  |  | 42 | 12.6 |  |  | 276 | 2.4 |  |
| **Died between**  **age 10 and 19** | 169 | 1.9 |  | 11 | 2.7 | 0.086 |  | 13 | 3.9 | 0.003 |  | 265 | 2.3 | 0.067 |
| **LTFU between**  **age 10 and 19** | 660 | 7.6 |  | 88 | 21.9 |  |  | 129 | 38.6 | <0.001 |  | 1134 | 9.8 | <0.001 |
| **Stunted at age 10** |  |  |  |  |  | <0.001 |  |  |  | <0.001 |  |  |  | <0.001 |
| Yes | 2524 | 28.9 |  | 72 | 18.0 |  |  | 8 | 2.4 |  |  | 3426 | 29.6 |  |
| No | 4300 | 49.2 |  | 232 | 57.9 |  |  | 3 | 0.9 |  |  | 6728 | 58.2 |  |
| Missing | 1913 | 21.9 |  | 97 | 24.2 |  |  | 323 | 96.7 |  |  | 1412 | 12.2 |  |
| **Wasted at age 10** |  |  |  |  |  | <0.001 |  |  |  | <0.001 |  |  |  | <0.001 |
| Yes | 733 | 8.4 |  | 34 | 8.5 |  |  | 11 | 3.3 |  |  | 1096 | 9.5 |  |
| No | 6288 | 72.0 |  | 245 | 61.1 |  |  | 0 | 0.0 |  |  | 8952 | 77.4 |  |
| Missing | 1716 | 19.6 |  | 122 | 30.4 |  |  | 212 | 63.5 |  |  | 1518 | 13.1 |  |
| **CD4 count at 10** |  |  |  |  |  | <0.001 |  |  |  | <0.001 |  |  |  | <0.001 |
| <250 | 569 | 6.5 |  | 4 | 1.0 |  |  | 0 | 0.0 |  |  | 453 | 3.9 |  |
| >250 | 4712 | 53.9 |  | 238 | 59.4 |  |  | 4 | 1.2 |  |  | 4638 | 40.1 |  |
| Missing | 3456 | 39.6 |  | 159 | 39.7 |  |  | 330 | 98.8 |  |  | 6476 | 56.0 |  |

*Chi-square tests, comparison between excluded groups A, B and C versus included population.

ART=antiretroviral therapy, HAZ= Height-for-Age Z-score, LTFU=Lost-To-Follow-Up.

Imputation 2

Imputation 3


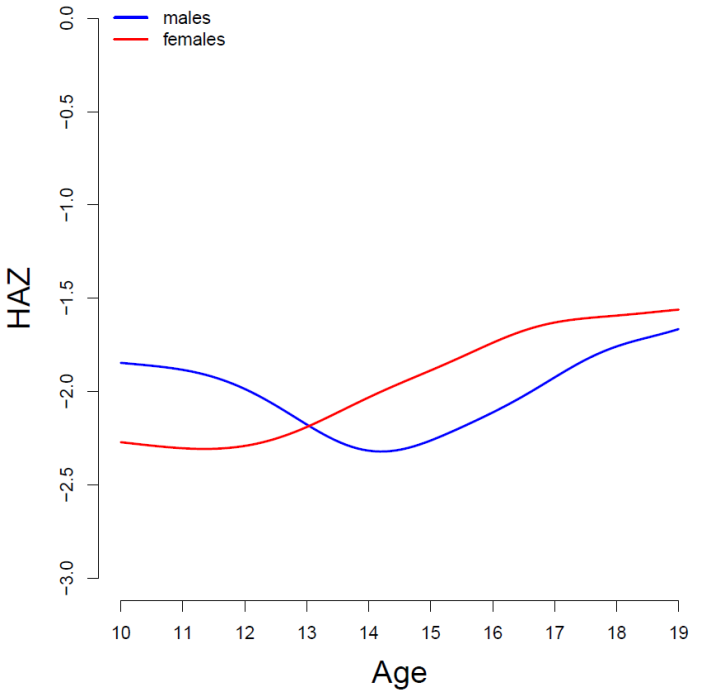

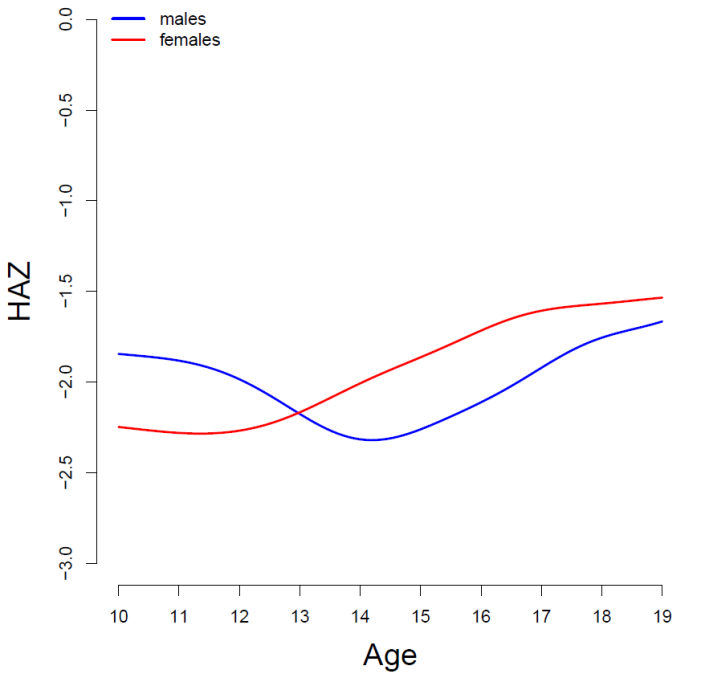


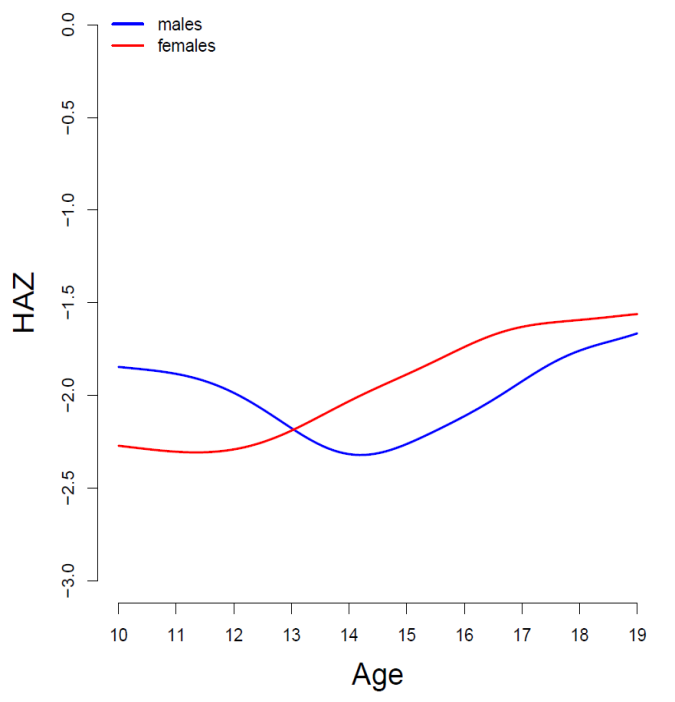


Imputation 5


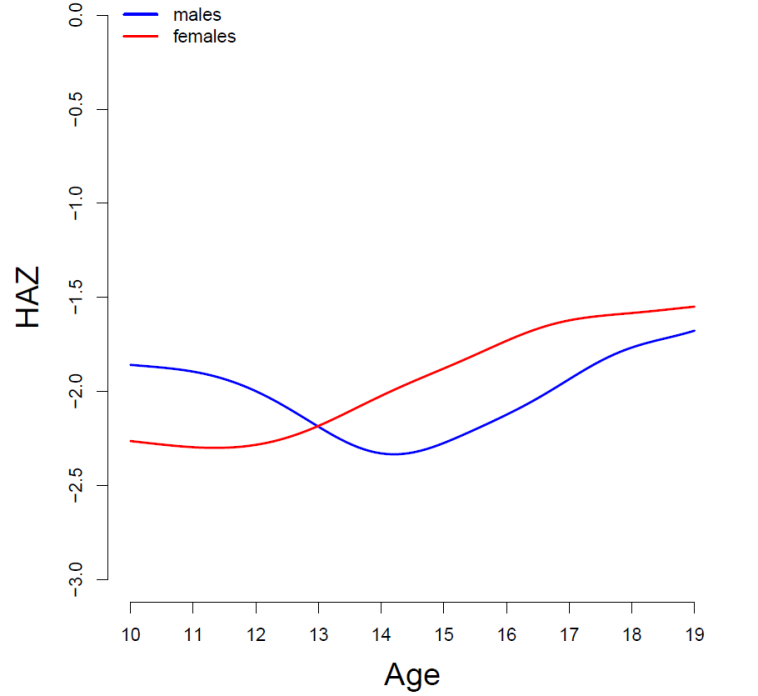


Imputation 4

Figure S1: Penalized splines for the association of age with HAZ for the 2-5 imputed data sets.

**
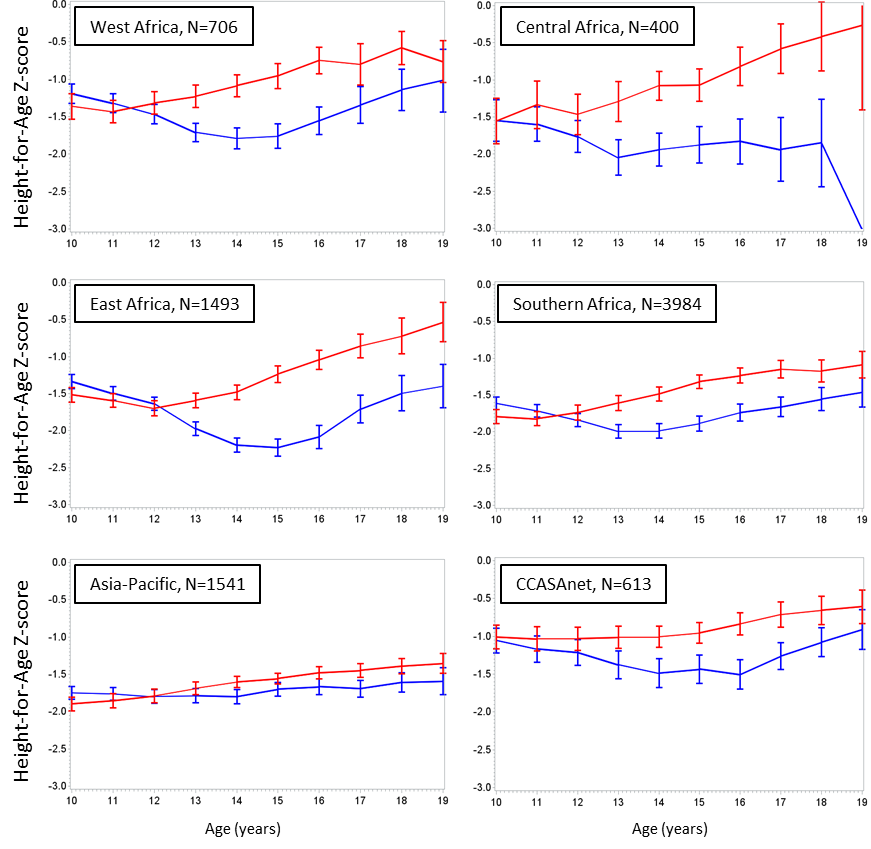
**

Figure S2: Mean Height-for-Age Z-score (HAZ) evolution for males (blue) and females (red) during adolescence, trends by regions using raw data. IeDEA global pediatric collaboration, 2003-2016
